# Facile Bench-Top Fabrication of Enclosed Circular Microchannels Provides 3D Confined Structure for Growth of Prostate Epithelial Cells

**DOI:** 10.1371/journal.pone.0099416

**Published:** 2014-06-19

**Authors:** Monika E. Dolega, Jayesh Wagh, Sophie Gerbaud, Frederique Kermarrec, Jean-Pierre Alcaraz, Donald K. Martin, Xavier Gidrol, Nathalie Picollet-D’hahan

**Affiliations:** 1 Univ. Grenoble Alpes, iRTSV-BGE, Grenoble, France; 2 CEA, iRTSV-BGE, Grenoble, France; 3 INSERM, BGE, Grenoble, France; 4 UJF-Grenoble 1, CNRS, TIMC-IMAG UMR 5525 (SyNaBi), Grenoble, France; Casey Eye Institute, United States of America

## Abstract

We present a simple bench-top method to fabricate enclosed circular channels for biological experiments. Fabricating the channels takes less than 2 hours by using glass capillaries of various diameters (from 100 µm up to 400 µm) as a mould in PDMS. The inner surface of microchannels prepared in this way was coated with a thin membrane of either Matrigel or a layer-by-layer polyelectrolyte to control cellular adhesion. The microchannels were then used as scaffolds for 3D-confined epithelial cell culture. To show that our device can be used with several epithelial cell types from exocrine glandular tissues, we performed our biological studies on adherent epithelial prostate cells (non-malignant RWPE-1 and invasive PC3) and also on breast (non-malignant MCF10A) cells We observed that in static conditions cells adhere and proliferate to form a confluent layer in channels of 150 µm in diameter and larger, whereas cellular viability decreases with decreasing diameter of the channel. Matrigel and PSS (poly (sodium 4-styrenesulphonate)) promote cell adhesion, whereas the cell proliferation rate was reduced on the PAH (poly (allylamine hydrochloride))-terminated surface. Moreover infusing channels with a continuous flow did not induce any cellular detachment. Our system is designed to simply grow cells in a microchannel structure and could be easily fabricated in any biological laboratory. It offers opportunities to grow epithelial cells that support the formation of a light. This system could be eventually used, for example, to collect cellular secretions, or study cell responses to graduated hypoxia conditions, to chemicals (drugs, siRNA, …) and/or physiological shear stress.

## Introduction

Model systems that recreate the architectural features observed in tissues and in tumours are of prime interest to study morphogenesis and carcinogenesis. Indeed in order to better mimic the reality of tissues compared to standard 2D culture, a growing number of 3D cell culture devices are being introduced to provide controlled mechanical, chemical and biological cues [1]. Numerous approaches have emerged that are aimed to control the spatial and temporal properties of the cell microenvironment (e.g. stiffness, 3D structure, micropatterning, shear stress). Endothelial cells cultured within a chamber capable of applying physiological shear stresses are induced to differentiate due to stimulation of specific integrin/endothelial cell-mediated signalling cascades [2]. Also, epithelial cells cultured on soft extracellular matrix gels organize themselves into polarized structures that strongly resemble functional tissue in vivo [3], [4]. For in vitro studies of vascular tissue, lab-on-chip (LOC) systems [5] that incorporate a unique 3D and dynamic microenvironment with high spatiotemporal precision provide a physiologically relevant way to reproduce vascular tissues.

However, despite the high prevalence of life-threatening diseases and cancers that affect exocrine glands, there are fewer reports of LOC systems to investigate exocrine ductal/acinar systems. We report here a microfluidics-based system that is simple to fabricate and provides 3D scaffolds that mimic epithelial-lined ductal systems of glandular tissues.

Microfluidics in general provides advantages such as *(i)* manipulation of liquids and objects at the microscale, *(ii)* high precision in controlling flow in low Reynolds number regimes (<<1), and *(iii)* facilitation of high-throughput experimentation by on chip parallelization and greatly reduced volume of expensive reagents and number of cells. Moreover, microfluidic systems have already allowed multiple biological studies including protein crystallization [6], collection of cellular secretions [7], blood circulation [8], angiogenesis [9] and cellular co-cultures [10].

Fabrication of microfluidic devices usually is based on micromachining of polymers such as poly(methyl methacrylate) [11], polycarbonate [12] or soft lithography with the use of polydimethylsiloxane (PDMS) [13]. The first technique requires the use of a milling machine and additional chemical treatments to bond different surfaces, while the latter relies on the use of highly equipped and specialized clean room facilities. To date, the most common profile of fabricated microchannels is rectangular. However, that profile does not reflect biological reality and more particularly the rheology of circular ducts (e.g. venules, arterioles, capillaries) nor tubular epithelial structures that are found in vivo. The rectangular microfabricated profile limits bio-applications and needs to be improved.

There are previous reports of the fabrication of microchannels in materials suitable for cell culture. Among them, Tien’s group used a similar method to fabricate circle channels in collagen hydrogels and investigates biology issues [14]–[16]. Wilson et al also described a method to obtain circular channels by combining mechanical micromilling with soft lithography [17]. Another approach relied on gas infusion into rectangular channels filled with a pre-polymerized agent [18], [19]. Despite the successful fabrication with the above techniques, they remain complex and time-consuming. A different approach to form circular channels was based on the moulding of nylon threads [20] or metal microwires [21] embedded inside a block of cross-linked PDMS and then removal. The technique did not require any bonding and created multichannel devices with circular channel diameters around 20 µm for microwires and larger than 50 µm for nylon threads. However, it was necessary to swell the PDMS with ethanol, triethylamine or chloroform for several hours before removal of threads or wires from the mould. That process therefore excludes the potential use of such systems for biological experiments due to the detrimental effect of organic swelling-solvents on living cells. A similar approach was also presented by Perry et al [22] for optically based flow cytometry studies of red blood cells. However, all these microfluidics systems are based on complex manufacturing processes that only a laboratory experienced in the field can reproduce. Furthermore, the previous systems lack biological validation with adhering cells and further characterization using specific markers of the organization or the morphology of the cells lining the walls of the tubes.

To overcome these limitations, we present a ready-to-use, simple and rapid method to fabricate single straight circular channels of various diameters for studies of adherent cells and for manipulation of the cellular microenvironment. Our technique takes about 2 hours and is based on the use of glass capillaries combined with corresponding needles that together serve as a mould for PDMS or hydrogel-based devices. We show here the biocompatibility of our system with various epithelial cells lines that grow stably inside the microchannels. Epithelial cells were chosen for three reasons. First, in 3D culture, they reproduce essential structural features of glandular epithelium in vivo, with the presence of a centrally-localized, hollow lumen and the polarization of cells surrounding that lumen. Second, we previously observed that some polyelectrolytes (PE)-terminating films preferentially modulate the 2D growth characteristics of prostatic cancerous cells [23]. Third, our previous data showed that prostatic cancerous cells are able to organize in 3D-confined tubular scaffolds [23]. In this study, we go further in showing the influence of the 3D microenvironment in tubes on epithelial cell morphology and proliferation by coating the microtubes either with a layer-by-layer assembled PE film or with Matrigel The PE film provides a tunable surface environment for external manipulation of cells and the Matrigel is typically used to mimic the complex extracellular environment found in many tissues. Our microchannel system allowed formation and access to an artificial lumen that allowed us to study cell morphogenesis and the events occurring during cells populating the lumen, such as in carcinogenesis. The microchannel ductal system allowed us to perform immunostaining and fluorescence microscopic observations of cells fixed inside the microchannel directly and without any special adjustment of the lumen.

## Materials and Methods

### 1. Fabrication of Microchannels

**Figure 1:**
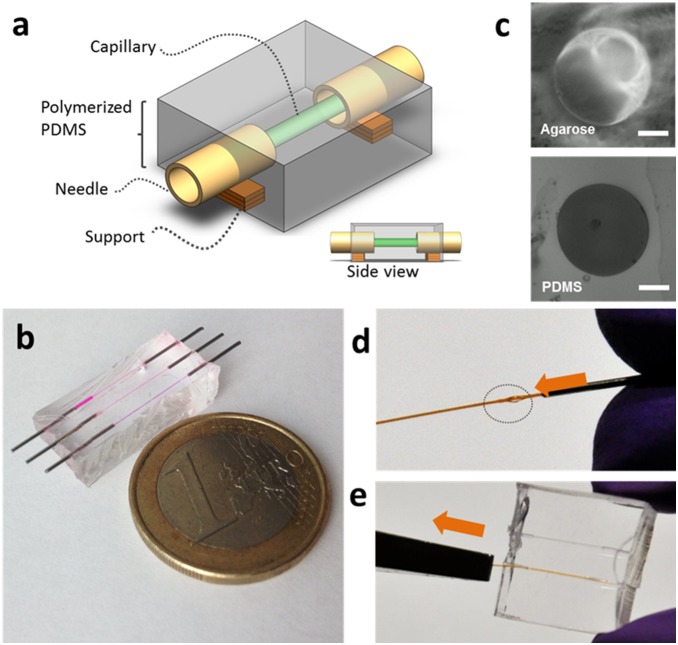
Microchannels fabrication process. Scheme of the device (**a**); Microphotograph of the device. One Euro coin added as a scale (**b**); Phase contrast pictures of cross section of channels fabricated in PDMS and agarose (**c**); Steps of fabrication (**d**) and (**e**). More detailed information provided in Figure S1. Scale bar 50 µm.

A step-by-step protocol to fabricate enclosed circular channels is presented in Figure 1 and is fully detailed in Figure S1 and Table S1 in File S1. The enclosed 3D cellular scaffolds were fabricated using glass capillaries as the mould to form circular microchannels (Figure 1). This bench-top fabrication procedure enables the preparation of multiple channels in parallel. For each experiment around 10 tubes were manufactured at one time within only two hours. Glass capillaries were mounted with needles which were immobilized by a drop of nail polish (Figure S1 in File S1). The capillary/needle assembly was positioned on two stacks to enable microscopy observations by controlling the distance between the channel and the surface of the PDMS. Degassed PDMS was poured into a petri dish to submerge the entire capillary architecture and then left to polymerize. After the PDMS had solidified it was removed from the petri dish and cut at the middle point of the needle to allow access to the assembly. Needles and subsequently, glass capillaries were gently removed with pliers (Figure S1 in File S1). This appropriate choice of capillaries (diameters and walls thicknesses) eliminates the need use ethanol or chloroform for smoothing PDMS prior to removing the capillaries. With these proper dimensions capillaries have also a very low tendency to break (less than 10% of capillaries, on n = 10 used in this work). New needles were inserted to allow injection of chemicals, nutrients and cells.

### 2. Coating of Microchannels

For biological assays, the inside of the PDMS microchannels was coated with a thin film of either polyelectrolytes or Matrigel (BD Biosciences, San Jose, CA, Ref 356231), which is widely used for 3D culture of epithelial cells due to its ability to promote specific cellular organization [3] and in case of endothelial cells for sprouting experiments. The thin film of polyectrolytes was achieved using Layer-by-Layer (LbL) assembly of the polyelectrolytes PSS (poly (sodium 4-styrenesulphonate; Sigma 243051)) and PAH (poly (allylamine hydrochloride; Sigma 283223)). They present different surface charges (polyanions for PSS and polycations for PAH) depending on the choice of the terminating layer. Also, the number of coatings modulates the mechanical and chemical properties. The process of deposition previously described [24] was adapted to this format of the microchannels (see Figure S2 in File S1). Since the polyelectrolytes bind Rhodamine B (Sigma, Ref R6626), the uniform coating of polyelectrolyte inside PDMS microchannels was confirmed by including Rhodamine (10 µg/ml) with the polyelectroytes. The microchannels were coated with the Rhodamine B/polyelectrolyte solution for 5 min at a flow rate of 1 ml.h^−1^ using a syringe pump.

The thin film of Matrigel was achieved by adapting a protocol for coating that is commonly used for 2D culture on glass and plastic substrates. We infused a diluted Matrigel (1∶40 in culture medium) into the microchannels for 30 minutes at RT and then washed the Matrigel solution out by culture medium.

### 3. Cell Lines and 2D Cell Culture

Adherent epithelial prostate cells (non-malignant RWPE-1 and invasive PC3) and breast (non-malignant MCF10A) were used. These cells form tree-like tubule structures with a hollow lumen when cultured in 3D [4], which mimic the *in vivo* glandular epithelial tissue architecture [25]. RWPE-1 (ATCC Ref. CRL-11609) and MCF10A (ATCC Ref. CRL-10317, gift from Odile Filhol, Inserm U1036, CEA Grenoble, France, [26]) cells respectively mimic normal prostate and breast epithelial cell behaviour as characterized by a well-established polarized morphology [4], whereas PC3 cells (ATCC Ref. CRL-1435) represent tumorigenic cells at a highly invasive stage of neoplastic transformation with the loss of acinar morphogenesis. PC3 cells (prostate carcinoma) were routinely cultured in RPMI Glutamax culture medium (Invitrogen, Ref. 61870-010) supplemented with 10% fetal calf serum (PAA, Ref. A15-101) and 1% penicillin/streptomycin (Invitrogen, Ref. 15140-122). RWPE-1 cells (non-neoplastic human prostate epithelial cells) were maintained in KSFM (Life Technologies, Carlsbad, CA, Ref. 17005-075) supplemented with 5 ng.mL^−1^ epidermal growth factor (EGF) and 50 µg.mL^−1^ bovine pituitary extract. For passaging, cells were washed with Dulbecco’s Ca^2+^/Mg-free PBS (D-PBS, Life Technologies, Ref. 14190) and incubated with 1 mL trypsin-EDTA (Lonza, Basel, CH, Ref. CC-5012, 0.25 mg.mL^−1^) for approximately 7 minutes, as previously described. MCF10A cells were maintained and cultured according to the protocol of Debnath et al [27]. All cells were routinely cultured in a humidified atmosphere at 37°C and 5% CO_2_. For all experiments cells were supplied every day with fresh culture medium.

### 4. 3D Cell Culture

Acinar morphogenesis assay was performed according to the previously published top-coat protocol [3]. Matrigel was thawed overnight and poured into either 4-well (160 µL of Matrigel, 500 µL of culture medium) or 8-well Labtek (90 µL of Matrigel, 250 µL of culture medium) plates on ice. Then, the plates were incubated for 30 minutes at 37°C in order to polymerise the Matrigel. After polymerizing the Matrigel cells were seeded in half the final volume (e.g. 5000 cells in each well of the 8-well Labtek plate) and allowed to adhere for approximately 45 minutes. The top coat layer of 8% Matrigel in culture medium was slowly poured over the attached cells. The culture medium was changed every other day.

### 5. Immunostaining in 2D and in 3D Culture

For 3D culture cells were fixed with 4% (v/v) paraformaldehyde (PFA) in PBS for 20 minutes and then washed once with PBS. 2D cultures were maintained until the cells attained 70% confluency and then were fixed with 4% (v/v) PFA in PBS for 20 minutes and then washed once with PBS. To prevent the nonspecific adsorption of antibodies, the cells were incubated with 0.1% BSA and 10% goat serum for 1 hour. The primary antibodies (anti-Giantin, Abcam ab24586, 1/500; anti-caspase 3 active, Millipore AB3623, 1/300; anti-beta4-integrin, Millipore MAB1964, 1/250; anti-centrosome, kind gift from M. Thery, CEA Grenoble, F) were dissolved in PBS^+^, Tween20 0.05%, and 5% goat serum. The cells were incubated for 1 hour with the appropriate antibody and then washed 4x with for a total of 45 minutes. The cells were then incubated with secondary antibody (Jackson, dilution 1/500 in PBS^+^, Tween20 0.05%, and 5% goat serum). Cells were subsequently washed 4 times for 15 minutes with PBS. Nuclei and actin were stained as described hereafter.

### 6. Static and Flow-based Infusion of Cells into Microchannels

Chemical dissociation of the cells was carried out by incubation for 5 min at 37°C in Trypsin-EDTA (Life Technologies, Ref. 25300-054) in calcium/magnesium-free PBS. Cells were then infused into microchannels using syringe/tubing connected to the device at high concentration of 3×10^6^ cells.mL^−1^ in order to infuse a sufficient number of cells into the microchannel. A syringe pump with an adjustable flow rate was the best choice to provide a gentle and controlled infusion. For static experiments, the chip with cells was positioned within a petri-dish and immersed in culture medium to prevent any evaporation from the channels. It is unnecessary to apply a constant flow of culture medium through the microchannel. For flow experiments, cells were infused into microchannels and kept in static conditions for 24 hours to promote satisfactory cellular adhesion. Stable and continuous flow was introduced by use of Fluigent pressure pumps (Fluigent, France). Pressurized containers with culture medium were set up within the CO_2_ and temperature controlled chamber. The flow rate was adjusted around 5–10 µL.h^−1^ (∼10 mbar) and adhesion and proliferation of cells were observed in time. All samples were kept in a humidified incubator at 37°C and 5% CO_2_.

### 7. Immunostaining and Viability Test in Microchannels

Phalloidin was used to identify cortical actin filaments, which follow the contours of the plasma membrane and hence provide a reasonable means to delineate the extent of the cell and its membrane. E-cadherin was used to detect cell-cell junctions. Immunostaining in microchannels was carried out by infusion with a syringe pump at room temperature throughout. RWPE-1 cells were plated in microchannels at a density of 6×10^6^ cells.mL^−1^ of culture. After the cells had formed a confluent layer (between 5–7 days), they were fixed for 20 minutes with 4% (v/v) PFA in a solution composed of 10% sucrose in cytoskeleton buffer (solution A). Then cells were washed with solution A and permeabilized for 3 minutes with 0.1% Triton TX-100 in solution A. The PFA auto-fluorescence was quenched by TBS 1X for 10 minutes, followed by washing with PBS for 30 minutes. Non-specific sites were blocked by exposure to 10% goat serum and 3% BSA in PBS and cells were incubated with the primary antibody for 1 hour. The primary antibody was E-Cadherin (Abcam, Ref. ab1416) diluted to 1/50 in 0.1% Tween-20 and 1% BSA in PBS. Cultures were then washed for 30 minutes with PBS and incubated with anti-mouse Cy3 conjugated secondary antibody (Jackson, Ref. 115-162-062) diluted to 1/1000 and Phalloidin FITC (Sigma, Ref. P5282) diluted to 1∶1000 in 0.1% Tween-20 and 1% BSA in PBS for 20 minutes. After washing for 30 minutes with PBS, nuclei were counterstained with Hoechst (Life Technologies, Ref. H-1399), diluted to 1∶7000, for 5 minutes. A last wash was performed for 10 minutes and Dako fluorescent mounting medium was infused manually.

Focal adhesion points were detected by labelling using Vinculin. For Vinculin immunostaining cells were pre-permeabilized for 40 seconds with Triton X-100 and fixed with 4% (v/v) PFA in PBS for 20 minutes and washed once with PBS. To prevent any nonspecific antibody adsorption cells were incubated with 0.1 BSA and 10% goat serum for 1 hour. Primary antibody against Vinculin (Sigma, Ref. V9131) at 1∶700 dilution (dissolved in PBS^+^, Tween 20 0.05%, and 5% goat serum) was incubated for 1 hour and followed by 4 PBS washes in total for 45 minutes. Cultures were then incubated with anti-mouse Cy5 (Jackson, dilution 1/500 in PBS^+^, Tween 20 0.05%, and 5% goat serum). Microchannels were subsequently washed 4 times for 15 minutes with PBS. Nuclei and actin were stained as described above. A Live/Dead Viability test was performed by use of Calcein AM (Molecular probes, C3099) and Propidium Iodide (Molecular Probes, V13245) according to the manufacturer’s protocols provided with the products.

### 8. Fluorescence Microscopy

The microchannels with cells inside were observed using the AxioImager Z1 Zeiss microscope with a 20x objective equipped with the straight Apotome module for z-stack acquisitions (one image in z-axis every 3 µm for a 150 µm-diameter tube). Images were recorded using an AxioCam MRm monochrome digital camera mounted to the microscope.

## Results and Discussion

Our motivation for this work was to develop polyelectrolyte 3D scaffolds in order to create new cell-culture devices that more closely mimic the natural physiological ductal environment found in exocrine glands. The present work described in this manuscript provides a detailed protocol for bench-top rapid fabrication of circular channels for 3D cell culture (Figure 1 & Figure S1 in File S1). The novelty of our system is based on its simplicity (Figure 1a). The fabrication process provides a high rate of success (nearly 90%) by the use of glass capillaries with thick walls (Table S1 in File S1), which are recommended to overcome potential for capillary breakage. Our process is cheap since it required low-cost materials, products and consumables (glass capillaries, needles, polymers). Therefore it is suited to parallelization. For convenience, about 10 tubes were manufactured at one time in this work to study different conditions but in fact, there is no limitation except the size of the support we are using. Therefore costs for performing these experiments in a larger scale setup are not more critical than for single experiments. This advantage opens the avenue towards further analysis (dose-response assays for instance) supported by an extensive statistical analysis according to specific needs. Compared to other existing methods, our fabrication of circular microchannels does not require the use of any complicated or specially adjusted moulds. The thick-called glass capillary is versatile since it can be used with different soft polymeric materials to form the microchannels, including agarose for example. Our method is limited to forming single microchannels, but which remains an advantage since it is much simpler compared to other existing methods and it provides more rapid fabrication. Although our method in its present description provides a very simple means to prepare microchannels to support ductal-like epithelial structures, it does provide a basis for future construction of more complex networked 3D microchannel scaffolds. Such future complex systems would include the capability for co-cultures of epithelial cells with stromal cells (fibroblasts, smooth muscle, endothelial cells) in order to reproduce functional tissues. Such a complex system could follow the same protocol of fabrication as the one presented here but would utilize another type of material, such as from within the hydrogels group. Although PDMS is easy to use, is biocompatible, has elastomeric properties, is flexible and perfectly transparent, it is only permeable to gases and does not allow for the diffusion of liquids. Applying our method to hydrogels, for example, would allow the possibility to *(i)* embed fibroblasts (or other type of cell) in the hydrogel and infuse epithelial cells into the microchannels so that the direct contact between two types of cells would be limited or *(ii)* infuse fibroblasts into the microchannels and culture them until confluence followed by the infusion of the second type of cells. One another possibility would be also to use sacrificial core and polyelectrolytes coating as previously demonstrated [34].

As a proof of concept for applying our method to such other biomaterials and to demonstrate the versatility of our technique for other types of temperature-dependent gels, we fabricated microchannels in agarose. Microchannels of 150 µm in diameter were fabricated in an agarose gel (3% w/v) following the same protocol as for PDMS (Figure 1b–c). Agarose is biologically inert and it can also be modified to promote cellular adhesion [28]. Cell culture, immunostaining and fluorescent microscopic observations can be performed directly without any extra manipulation. Moreover, the rounded-shape of our fabricated microchannels and the compatibility of our device with biopolymer coatings (e.g. Matrigel, LbL-assembled polyelectrolytes) allow extending bio-applications to 3D cell culture to flow-based assays which more closely mimic the physiological context of exocrine gland function.

Since cells do not adhere well to untreated PDMS, several treatments can be applied including plasma treatment and further amine functionalization [29], protein absorption or boiling water [30] to promote cellular adhesion. We chose to modify the microchannels by the simple method of layer-by-layer assembly of alternating polyanionic (PSS) and polycationic (PAH) materials [31]. This assembly produces a different surface charge that depends on the choice of the terminated PE layer, a modulation of the mechanical properties of the surface that depends of the number of layers assembled, and is also known to produce a surface that is compatible with cell adhesion [24]. Moreover we previously demonstrated quantitatively that PSS and PAH coatings alter the growth, attachment and spreading of prostate epithelial cells [23].

In this study we used three different types of human epithelial cells types to validate that the microchannels constitute a proper 3D microenvironment. In 3D culture, normal cells form indeed tubules, organized as a tree-like structure mimicking the in vivo glandular epithelial tissue architecture. Tubules connect terminal ductal lobular units, namely acini that are the smallest functional units of the prostate and the breast. Acini and tubules are formed by lumen-enclosing monolayers of cohesive cells and are respectively spherical and cylindrical. These cells models are routinely used in our lab to investigate 3D constructs in bulk Matrigel cultures (Figure S2 in File S1) and do properly differentiate into normal, hollow and well differentiated acini (for RWPE1 and MCF10A) [3]. As expected, PC3 cells in our hands form non-differentiated tumor-like spheroids (data not shown). Moreover these models are known to exhibit an exocrine activity, such as RWPE1 for example previously shown to secrete PSA (Prostate Specific Antigen) [35]–[36]. However, similar to other labs working with such bulk Matrigel 3D cultures, it is not possible to access in real-time the secretions from acini/ductal structures that form spontaneously in the bulk Matrigel.

**Figure 2:**
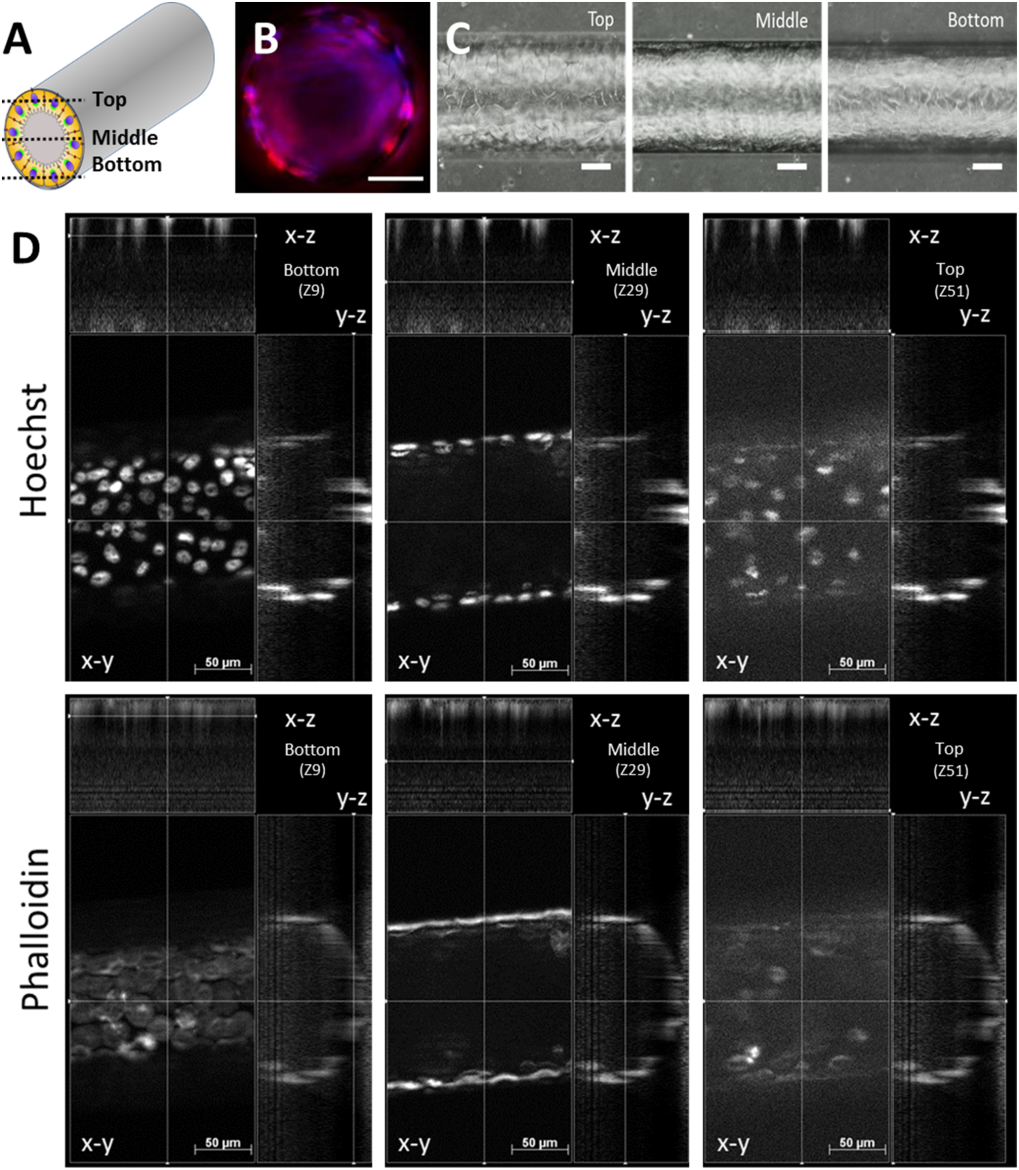
Epithelial cellular organization and proliferation inside fabricated microchannels. **A.** Schematic representation of an epithelial cell monolayer covering the inner wall of the circular channels fabricated in polydimethylo-siloxane (PDMS). **B.** Cross-section of a microchannel formed in PDMS and coated with Matrigel. Cells infused into channels adhere to 3D-confined scaffolds and form a confluent layer (Actin in red, nuclei in blue). Bar 50 µm. **C.** Organization of MCF10A cells forming a confluent monolayer after 5 days of growth inside a Matrigel-coated tube (diameter 150 µm). Phase contrast images. Note that the focus of each image is at different plane (top, middle and bottom of the tube as indicated in A.). Scale bar = 100 µm. **D.** Apotome Z-stack of PC3 cells on PSS substrate stained with Hoechst (top panel) and Phalloidin (lower panel). Separate-channel images are shown and three positions of the tubes (as indicated in A) are viewed.

**Figure 3:**
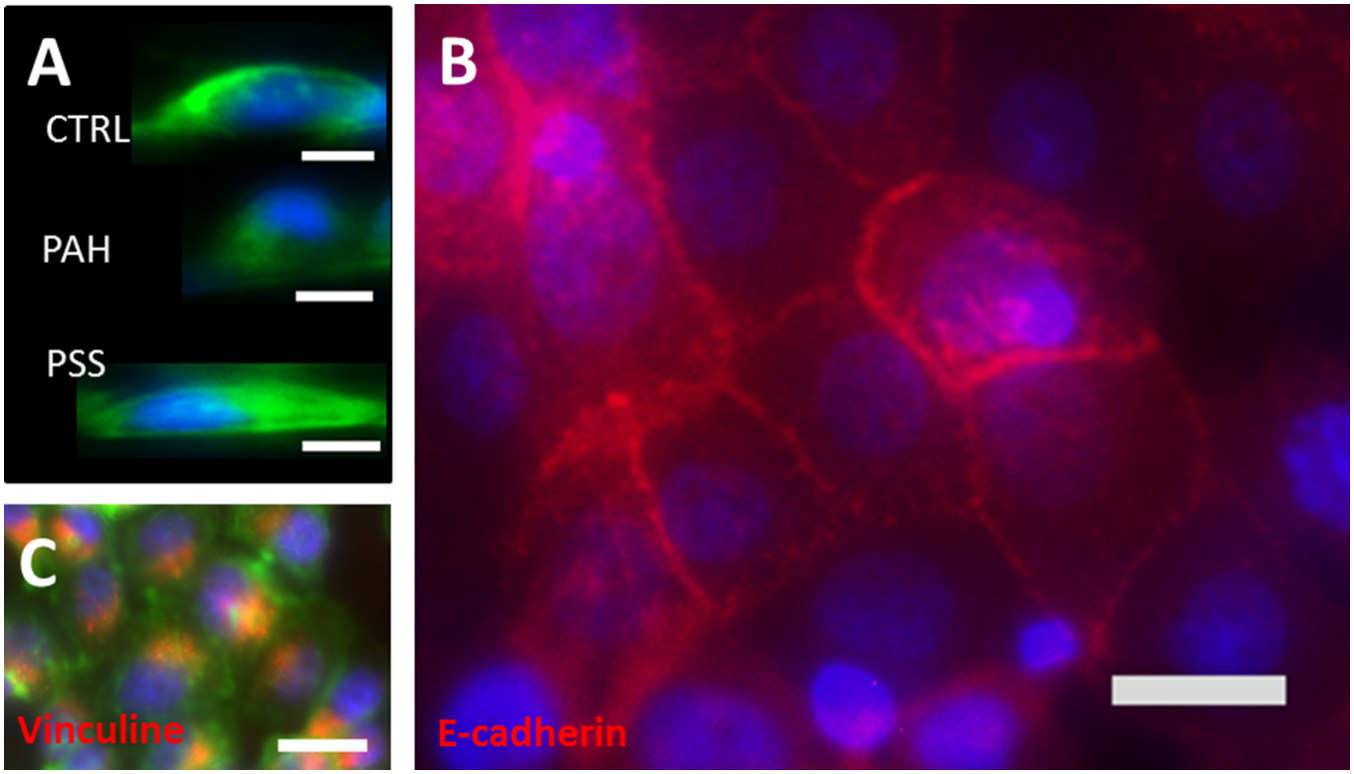
Epithelial cellular morphology and adhesion inside fabricated microchannels. **A.** Focus at midline of a circular channel provides direct view of RWPE1 cell and nuclei shape that vary according to PE coating. Bar 10 µm. **B.** Membrane junction structures of RWPE1 cells adhering in tubes. Expression of E-Cadherin (red) reveal intercellular junctions formed in the confluent monolayer of cells lining the inner wall of the tubes (240 µm diameter). Nuclei in blue (DAPI). Scale bare 25 µm. **C.** Adhesion of PC3 cells in Matrigel coated channels of 150 µm in diameter. Focal adhesion points (Vinculin) in red, nuclei in blue (DAPI) and actin in green (Phalloidin). Scale bare 25 µm.

Our results in this report demonstrate *i)* conditions required for cells to attach and grow as a confluent and viable monolayer on the walls of the microchannel (Figure 2A–B) just as standard 2D cell culture on dishes (Figure S2 in File S1) and *ii)* the formation of a light in the centre of the microchannels, for example as a basis for creating acini/ductal structures for the collection of secretions in real-time. Our phase contrast results show that all cell lines adhered and proliferated inside fabricated microchannels of 150 µm of diameter and formed a confluent monolayer covering the inner wall of the microchannel in static conditions after approximately 5 days (Figure 2C), as also demonstrated with simultaneous staining for Phalloidin and nuclei observed with confocal imaging (Figure 2D). The Apotome z-stack in Figure 2D provides a large-area 2D projection of the entire microtube. Focal adhesion sites were identified by Vinculin immunostaining and are shown here for PC3 as a representative result consistent with what we observed on all our models (Figure 3). Vinculin is a cytoskeletal protein associated with cell-cell and cell-matrix junctions, where it is thought to function as one of several interacting proteins involved in anchoring F-actin to the plasma membrane. We show that a microchannel coated with either a single PSS layer or a layer of Matrigel promotes cellular adhesion and proliferation of epithelial cells when observed with phase contrast and fluorescent microscopy (Figure 2). The PE deposition inside microchannels was confirmed by addition of Rhodamine B into polyelectrolyte solution and observation under microscope (Figure S3 in File S1). By contrast to PSS and Matrigel, a single layer of PAH slowed down cell proliferation and spreading, similar to a previous report [23]. It is commonly assumed that positively charged surfaces promote cell adhesion and proliferation [32]. However, PAH-terminal films support cell culture selectively depending on the cell line; for example, positively for neuronal cells while negatively for skeletal muscle cells [33]. The observed limited adhesion of epithelial cells in PAH-coated microchannels was also illustrated by the height of the cell and the position of nucleus (Figure 3A) as compared to PSS (spread cellular profile) and uncoated PDMS surface (rounded shaped cells). The ultimate commitment of a cell to adhere, differentiate, proliferate or migrate is a well-coordinated response to its molecular interaction with the components of the extracellular matrix (ECM) and surrounding cells. To further characterize cellular structures formed in the microchannels, expression of E-Cadherin was observed by the epithelial cells (Figure 3B). The expression of E-cadherin, a marker of lateral membrane domains, shows that our ex vivo microchannel provides an environment for the proper architecture and differentiated function of epithelial tissue. E-cadherin is also often used to quantify epithelial to mesenchymal transition and hence was chosen here to validate that our cells do not undergo phenotypic alteration. The luminal expression of Golgi in 3D (Figure S2 in File S1) also demonstrated that acinar differentiation was in line with cell polarization. This polarity was observed both in 2D and in 3D with the luminal expression of Giantin and of the centrosome (Figure S2 in File S1). This polarized organization of cells that we could observe in 2D does not mean that cells in 2D will undergo the same functional phenotype that they do in 3D. It is indeed well-known that the function of secretory cells such as breast epithelial cells [37] changes dramatically from the classical 2D culture plates to even Matrigel cultures. Also, the responses of tumor tissues to anti-cancer drugs are radically different between 2D and 3D cultures [38].

**Figure 4:**
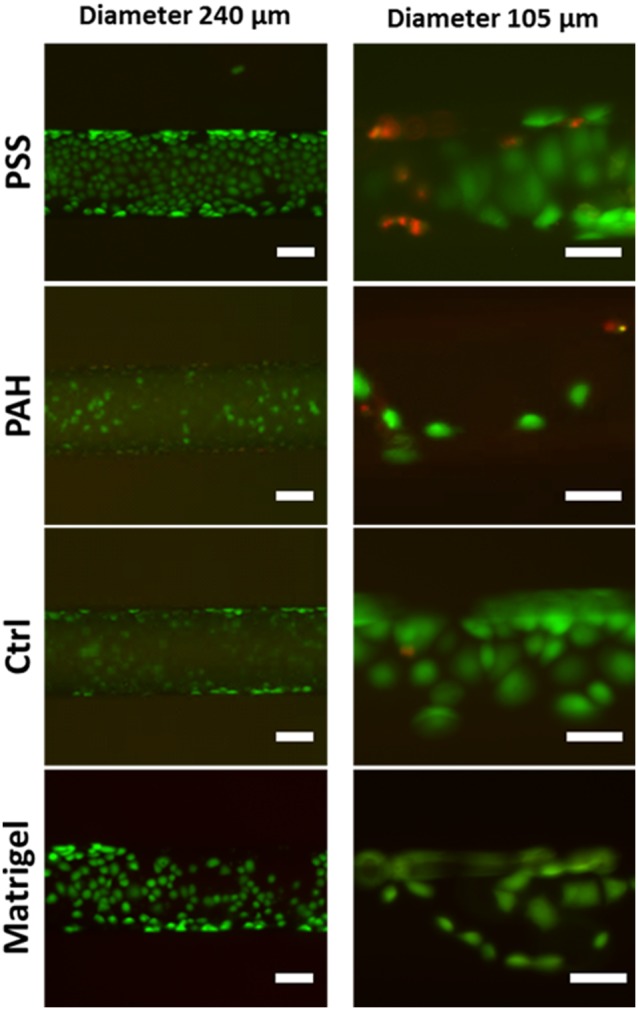
PC3 cell viability in microchannels. Calcein AM/propidium iodide test on PC3 cells in tubes of 105 µm (right) and 240 µm (left) coated with polyelectrolytes (PAH or PSS) and Matrigel. Note the limited adhesion and proliferation of cells in channels coated with PAH and non-coated (CTRL). In 105 µm channels we observe increased cellular death and the same effect of the type of coating. Scale bar 50 µm.

To assess the biocompatibility of the fabricated scaffolds we performed viability assays on PC3 cells cultured in microchannels of various diameters (Figure 4). For those assays we chose the smallest diameter of the channel to be 105 µm, since this is a dimension that is similar to the acinar and tubular structures existing in vivo. For comparison, we utilised a microchannel of 240 µm in diameter as our reference of stable proliferation of cells inside microchannels. We based our viability tests on two parameters; the intercellular esterase activity as indicated by calcein AM and plasma membrane integrity as verified by propidium iodide. By observation of the live/dead cell ratio we could find the best conditions for the cell culture inside the microchannels. We tested several coatings (PAH, PSS, no coating and Matrigel coating) and we found the best to be the PSS and Matrigel coatings (Figure 4). As previously observed and quantified, we confirm that PAH limits cellular adhesion and proliferation in comparison to the PSS and Matrigel coatings which provided cellular organization within the tubes [23]. Moreover we verified the importance of the diameter of the channels on two representative dimensions (240 µm and 150 µm). In static conditions we noticed that the cellular proliferation and viability are limited in 105 µm microchannels, as compared to the microchannels of 240 µm diameter where the growth of cells varied only according to the type of coating (Figure 4). We explain these results by the limited diffusion of nutrients in the smaller channels. In 105 µm channels the effect of surface coating also plays important role; however, we observed that cells proliferated mostly at the terminal ends of the channels which suggests limited diffusion of fresh nutrients into the channel. The observed lack of growth might also relate to the lack of oxygen. Interestingly our system could be useful to create defined and gradual hypoxia conditions without any need for special hypoxic incubator. This limitation can be overcome when perfusing channels with a continuous flow of nutrients without affecting cell viability and adhesion. Indeed we did not observe any cellular detachment due to the presence of the flow (see Movie S1 in File S1). As expected and observed using time-lapse microscopy, detachment during mitosis obviously occurs inside channels but the daughter cells following division consequently re-attach to the substrate. The precise and careful adjustment of the flow rate and pressure values during cell infusion and culture has prevented cells removal during division. Therefore, cells during mitotic detachment are capable to maintain in the channel and adhere. All observations taken together, the size of channels does not hamper the proper cellular division, adhesion and organization inside cylindrical tubules. Further experiments are under progress in our group to finely investigate the effect of flow on cellular function (secretions). In the current study we succeeded in validating the feasibility, usefulness and biocompatibility of our microchannels and their adequacy with our epithelial models. Detecting secretions and demonstrating a functional assay on exocrine cells represent our ultimate goal. In this context microsystems offer a distinct advantage with the high volume to surface ratio of capillaries. While small scale is responsible for limited cell growth in static conditions due to the quick starvation (as shown for small channels of 100 µm diameter), any tiny secretions are more concentrated in a small volume which simplifies the detection. To that end, the proposed system in the future would be easily combined with mass spectrometry techniques for secretomics-based analysis.

## Conclusions

Our technique provides a simple, low cost and versatile means to fabricate circular-shaped microchannels that are well-adapted for bio-applications. Epithelial cell behaviour can be modulated in microchannels by altering the surface charge of the microenvironment (PE) or by mechanical constraint (i.e. by adjusting the diameter of the microchannel). Our method produces a device that is easily combined with microfluidic flow systems for extensive studies of cell culture in dynamic environments and of further 3D tubulogenesis models. We believe that these circular microchannels can successfully serve for diverse epithelial cell types from exocrine tissues but also for endothelial and blood flow experiments, cellular secretion collection and co-culture systems, monitoring cellular processes in 3D and studying the dynamic flow effects on cell function. Moreover, it is a simple and rapid fabrication process that could be easily integrated in any biological laboratories.

## Supporting Information

File S1 Contains Figure S1, A serie of photographs detailing the fabrication process. Figure S2, Epithelial cellular organization in 2D and 3D. Figure S3, Rhodamine staining of polyelectrolyte-coated PDMS channels. Table S1, Guide for choice of capillaries and needles for the microfabrication process.
(DOCX)

Movie S1 Presents the monolayer formation of MCF10A cells within first 15 hours culture with adjusted flow of culture media.
(DOCX)

## References

[1] Lutolf MP, Hubbell JA (2005) Synthetic biomaterials as instructive extracellular microenvironments for morphogenesis in tissue engineering. Nature Biotechnology 23: 47–55. 10.1038/nbt1055 15637621

[2] Riha GM, Lin PH, Lumsden AB, Yao QZ, Chen CY (2005) Roles of hemodynamic forces in vascular cell differentiation. Annals of Biomedical Engineering 33: 772–779. 16078617 10.1007/s10439-005-3310-9

[3] Dolega ME, Allier C, Vinjimore KS, Gerbaud S, Kermarrec F, et al (2013) Label-free analysis of prostate acini-like 3D structures by lensfree imaging. Biosens Bioelectron 49c: 176–183. 10.1016/j.bios.2013.05.001 23747358

[4] Debnath J, Brugge JS (2005) Modelling glandular epithelial cancers in three-dimensional cultures. Nature Reviews Cancer 5: 675–688. 16148884 10.1038/nrc1695

[5] Whitesides GM (2006) The origins and the future of microfluidics. Nature 442: 368–373. 16871203 10.1038/nature05058

[6] Dolega ME, Jakiela S, Razew M, Rakszewska A, Cybulski O, et al (2012) Iterative operations on microdroplets and continuous monitoring of processes within them; determination of solubility diagrams of proteins. Lab on a Chip 12: 4022–4025. 22868285 10.1039/c2lc40174f

[7] Huang N-T, Chen W, Oh B-R, Cornell T, Shanley TP, et al (2012) An integrated microfluidic platform for in situ cellular cytokine secretion immunophenotyping. Lab on a Chip 12: 4093–4101. 22892681 10.1039/c2lc40619e PMC3508001

[8] Shevkoplyas SS, Gifford SC, Yoshida T, Bitensky MW (2003) Prototype of an in vitro model of the microcirculation. Microvascular Research 65: 132–136. 12686171 10.1016/s0026-2862(02)00034-1

[9] Song JW, Munn LL (2011) Fluid forces control endothelial sprouting. Proc Natl Acad Sci USA 108: 15342–15347. 21876168 10.1073/pnas.1105316108 PMC3174629

[10] Rao N, Evans S, Stewart D, Spencer KH, Sheikh F, et al (2013) Fibroblasts influence muscle progenitor differentiation and alignment in contact independent and dependent manners in organized co-culture devices. Biomedical Microdevices 15: 161–169. 22983793 10.1007/s10544-012-9709-9 PMC3537877

[11] Brown L, Koerner T, Horton JH, Oleschuk RD (2006) Fabrication and characterization of poly (methylmethacrylate) microfluidic devices bonded using surface modifications and solvents. Lab on a Chip 6: 66–73. 16372071 10.1039/b512179e

[12] Martin PM, Matson DW, Bennett WD, Hammerstrom DJ (1998) Fabrication of plastic microfluidic components. Proceedings microelectronic Engineering, Santa Clara, Ca 41/42: 493–496.

[13] McDonald JC, Whitesides GM (2002) Poly(dimethylsiloxane) as a material for fabricating microfluidic devices. Accounts of Chemical Research 35: 491–499. 12118988 10.1021/ar010110q

[14] Golden AP, Tien J (2007) Fabrication of microfluidic hydrogels using molded gelatin as a sacrificial element. Lab on Chip 7 (6): 720–5. 10.1039/b618409j 17538713

[15] Price GM, Tien J (2011) Methods for forming human microvascular tubes in vitro and measuring their macromolecular permeability. Methods Mol Biol 671: 281–93. 20967637 10.1007/978-1-59745-551-0_17

[16] Wrong KH, Chan JM, Kamm RD, Tien J (2012) Microfluidic models of vascular functions. Annu Rev Biomed Eng 14: 205–30. 22540941 10.1146/annurev-bioeng-071811-150052

[17] Wilson ME, Kota N, Kim Y, Wang Y, Stolz DB, et al (2011) Fabrication of circular microfluidic channels by combining mechanical micromilling and soft lithography. Lab on a Chip 11: 1550–1555. 21399830 10.1039/c0lc00561d

[18] Fiddes LK, Raz N, Srigunapalan S, Tumarkan E, Simmons CA, et al (2010) A circular cross-section PDMS microfluidics system for replication of cardiovascular flow conditions. Biomaterials 31: 3459–3464. 20167361 10.1016/j.biomaterials.2010.01.082

[19] Abdelgawad M, Wu C, Chien WY, Geddie WR, Jewett MAS, et al (2011) A fast and simple method to fabricate circular microchannels in polydimethylsiloxane (PDMS). Lab on a Chip 11: 545–551. 21079874 10.1039/c0lc00093k

[20] Verma MKS, Majumder A, Ghatak A (2006) Embedded template-assisted fabrication of complex microchannels in PDMS and design of a microfluidic adhesive. Langmuir 22: 10291–10295. 17107035 10.1021/la062516n

[21] Jia Y, Jiang J, Ma X, Li Y, Huang H, et al (2008) PDMS microchannel fabrication technique based on microwire-molding. Chinese Science Bulletin 53: 3928–3936.

[22] Perry H, Greiner C, Georgakoudi I, Cronin-Golomb M, Omenetto FG (2007) Simple fabrication technique for rapid prototyping of seamless cylindrical microchannels in polymer substrates. Review of Scientific Instruments 78 (4): 044302. 10.1063/1.2719626 17477682

[23] Picollet-D’hahan N, Gerbaud S, Kermarrec F, Alcaraz JP, Obeid P, et al (2013) The modulation of attachment, growth and morphology of cancerous prostate cells by polyelectrolyte nanofilms. Biomaterials 34 (38): 10099–108. 10.1016/j.biomaterials.2013.08.093 24060421

[24] Ting JHY, Haas MR, Valenzuela SM, Martin DK (2010) Terminating polyelectrolyte in multilayer films influences growth and morphology of adhering cells. IET Nanobiotechnology 4: 77–90. 20726674 10.1049/iet-nbt.2009.0016

[25] Mailleux AA, Overholtzer M, Brugge JS (2008) Lumen formation during mammary epithelial morphogenesis: insights from in vitro and in vivo models. Cell Cycle 7: 57–62. 18196964 10.4161/cc.7.1.5150

[26] Deshiere A, Duchemin-Pelletier, Spreux E, Ciais D, Combes F, et al (2013) Unbalanced expression of CK2 kinase subunits is sufficient to drive epithelial-to-mesenchymal transition by Snail1 induction. Oncogene 32: 1373–1383. 22562247 10.1038/onc.2012.165

[27] Hebner C, Weaver VM, Debnath J (2008) Modeling morphogenesis and oncogenesis in three-dimensional breast epithelial cultures. Annual Review of Pathology-Mechanisms of Disease 3: 313–339. 10.1146/annurev.pathmechdis.3.121806.151526 18039125

[28] Su Y, Chu B, Gao Y, Wu C, Zhang L, et al (2013) Modification of agarose with carboxylation and grafting dopamine for promotion of its cell-adhesiveness. Carbohydrate Polymers 92: 2245–2251. 23399284 10.1016/j.carbpol.2012.12.003

[29] Glass NR, Tjeung R, Chan P, Yeo LY, Friend JR (2011) Organosilane deposition for microfluidic applications. Biomicrofluidics 5(3): 36501–365017. 22662048 10.1063/1.3625605 PMC3364836

[30] Joong Yull P, Dongchan A, Yoon Young C, Chang Mo H, Takayama S, et al (2012) Surface chemistry modification of PDMS elastomers with boiling water improves cellular adhesion. Sensors and Actuators B (Chemical) 173: 765–771.

[31] Decher G, Hong JD, Schmitt J (1992) Buildup of ultrathin multilayer films by a self-assembly process: III. Consecutively alternating adsorption of anionic and cationic polyelectrolytes on charged surfaces. Thin Solid Films.

[32] Bledi Y, Domb AJ, Linial M (2000) Culturing neuronal cells on surfaces coated by a novel polyethyleneimine-based polymer. Brain Research Protocols 5: 282–289. 10906494 10.1016/s1385-299x(00)00024-6

[33] Dhir V, Natarajan A, Stancescu M, Chunder A, Bhargava N, et al (2009) Patterning of diverse mammalian cell types in serum free medium with photoablation. Biotechnology Progress 25: 594–603. 19334291 10.1002/btpr.150 PMC2966384

[34] Ting JHY (2008) Diabetic retinopathy: Economic evaluations and cellular functions. PhD thesis, University of Technology Sydney, Australia.

[35] Webber MM, Bello D, Kleinman HK, Hoffman MP (1997) Acinar differentiation by non-malignant immortalized human prostatic epithelial cells and its loss by malignant cells. Carcinogenesis 18: 1225–1231. 9214606 10.1093/carcin/18.6.1225

[36] Nicotera TM, Schuster DP, Bourhim M, Chadha K, Corral DA, et al (2009) Regulation of PSA Secretion and Survival Signaling by Calcium-Independent Phopholipase A(2)beta in Prostate Cancer Cells. Prostate 69: 1270–1280. 19475654 10.1002/pros.20968

[37] Vidi P-A, Bissell MJ, Lelievre SA (2013) Three-dimensional culture of human breast epithelial cells: the how and the why. Methods in molecular biology (Clifton, NJ) 945: 193–219. 10.1007/978-1-62703-125-7_13 PMC3666567 23097109

[38] Godugu C, Patel AR, Desai U, Andey T, Sams A, et al (2013) AlgiMatrix (TM) Based 3D Cell Culture System as an In-Vitro Tumor Model for Anticancer Studies. Plos One 8: 13. 10.1371/journal.pone.0053708 PMC3548811 23349734

