# Supplementary material for: Facile Bench-Top Fabrication of Enclosed Circular Microchannels Provides 3D Confined Structure for Growth of Prostate Epithelial Cells
Source: PLoS One. 2014 Jun 19;9(6):e99416. doi: 10.1371/journal.pone.0099416 (PMC4063722; doi:10.1371/journal.pone.0099416)
Supplement: File S1 — Contains Figure S1, A serie of photographs detailing the fabrication process. Figure S2, Epithelial cellular organization in 2D and 3D. Figure S3, Rhodamine staining of polyelectrolyte-coated PDMS channels. Table S1, Guide for choice of capillaries and needles for the microfabrication process. (DOCX) [file pone.0099416.s001.docx]

**Supplementary Information S1**


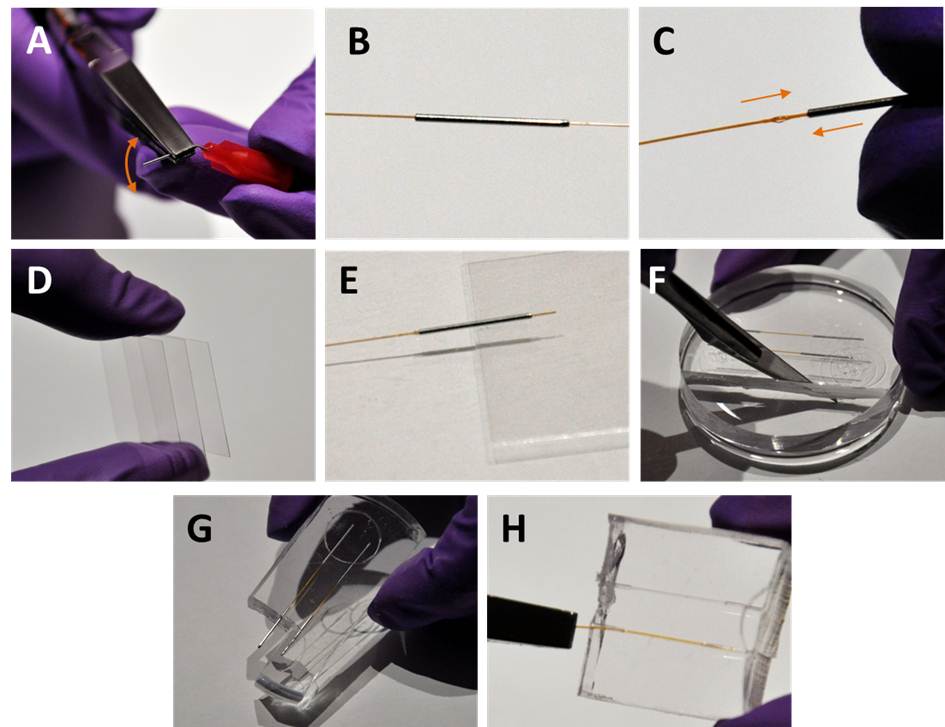


**Figure S1 in File S1. A serie of photographs detailing the fabrication process.** PDMS was prepared by extensive mixing of the elastomer with the curing agent in a 10:1 (wt/wt) ratio. During mixing air bubbles are introduced due to the viscosity of the solution and it is recommended to remove them by using a desiccator. Then the glass capillary is cut with scissors to a desired length, leaving an allowance of at least 1 cm to protrude from each side of the final construct. The needles are supplied with a plastic syringe connector that has to be cut carefully using a dremel tool or by hand with the use of pliers (**A**). Make sure that the metal part of needle has not been damaged or curved. Blow the needles with air to get rid of any inner contamination. The glass capillary is then aligned with the needle (**B**) so that the capillary protrudes from each end of the needle. To prevent any misalignment during further steps put a small drop of transparent nail polish on the surface of capillary at each end and slide the needle part so that the nail polish fills the space between the needle and the capillary (**C**). Triple glass cover slip towers are used to set up the distance between the channel and the surface of the petridish to ~550 µm (**D**). This step is crucial for further microscopic observations. In a 35 mm petridish (or other container of choice) support is inserted to keep the capillary/needle at controlled distance from the bottom (**E**). Degassed PDMS is then gently poured into a petri dish to completely cover the construct. The complete system is then placed in 65°C for 50 minutes to polymerize the PDMS. This process can be further accelerated with increased temperature. However, the plastic petridish would then need to be replaced with high temperature resistant container. The polymerized PDMS is then removed with embedded capillary/needle construct and the support from the petridish and cut out using a scalpel thoroughly in the middle point of the needle perpendicularly to the channel at both ends (**F-G**). The cut part of the PDMS are gently removed from the needles and with the help of pliers the needles are pulled out first then the capillary from the PDMS (**H**). To prevent biological contamination, the device is sterilized by baking in an oven at 120°C for 30 minutes.


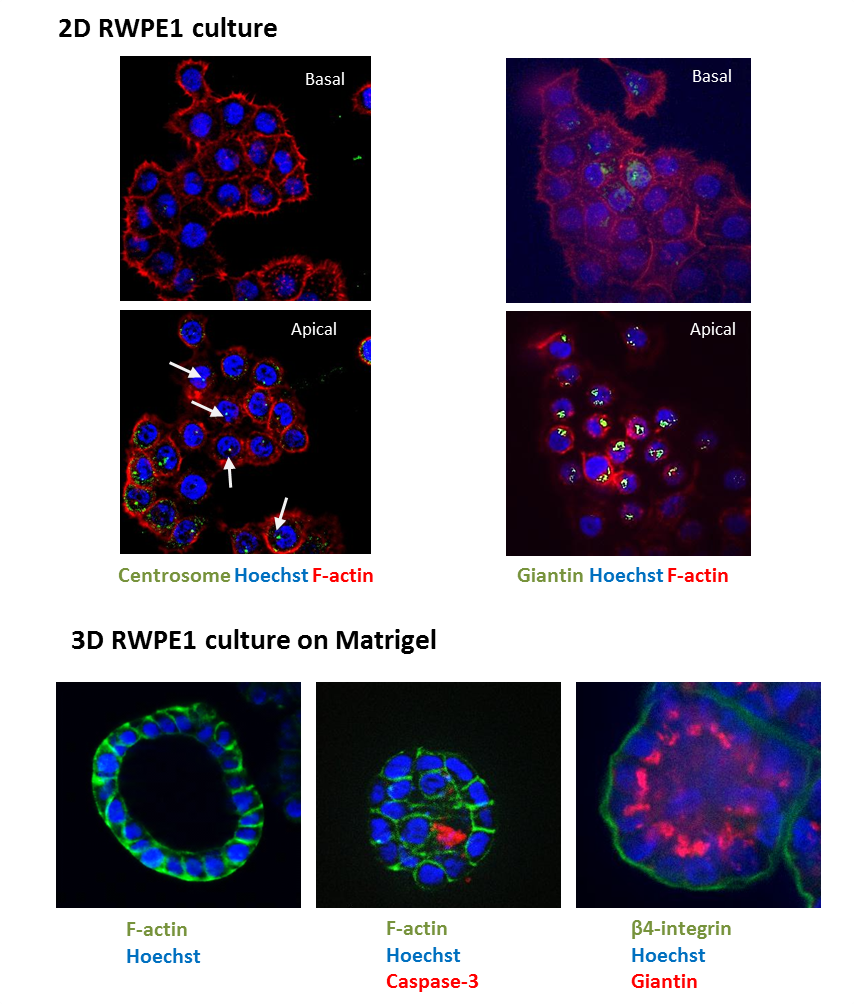


**Figure S2 in File S1. Epithelial cellular organization in 2D and 3D.**

*Top panel*: Immunostaining of nuclei (blue), F-actin (red) and centrosome (green, left panel) or Golgi (green, right panel) of RWPE1 cells after 72 hours culturing in 2D on plastic substrates. Cells were imaged by confocal at the interface cell membrane / surface (basal) and at the interface cell top / culture medium (luminal). The apical polarity is observed with centrosome staining (arrows) and Golgi staining. *Lower panel*: Immunostaining of nuclei (blue), F-actin (green) and caspase-3 (red, middle panel) and in the right panel, of Golgi (red) and B4-integrin (green) of RWPE1 cells culture in 3D Matrigel.


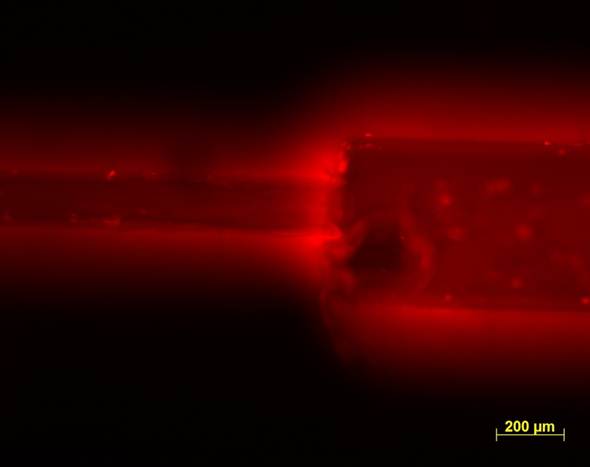


**Figure S3 in File S1.**  **Rhodamine staining of polyelectrolyte-coated PDMS channels.** Channels were coated by constant infusion of the particular polyelectrolyte for 20 minutes followed by washing with PBS. To deposit a multilayer film, polyanions and polycations were infused alternatively, and between the changes of polyelectrolyte solution the tubes were washed thoroughly with MilliQ-grade water (18 MΩ cm).

| Needle ref. (EFD Nordsen) | Needle ID | Needle OD | For channel | Capillary ref. (PolymicroTech.) |
| --- | --- | --- | --- | --- |
| PN7018395 | 200 µm | 420 µm | 150 µm, 150 µm | TSP040105, TSP002150-10M |
| PN7018345 | 250 µm | 520 µm | 150 µm,240 µm | TSP002150-10M, TSP100245 |
| PN7018272 | 410 µm | 720 µm | 363 µm | TSP005375-10M |
| PN7018233 | 510 µm | 820 µm | 435 µm | TSP320450 |

**Table S1 in File S1**. **Guide for choice of capillaries and needles for the microfabrication process.** Table S1 presents the dimensions of needles and capillaries that have been used for the experiments presented in this article. Depending on the desired channel size, a particular capillary and corresponding needle combination needs to be chosen. Therefore, Table S1 can serve as a guide for choosing a capillary/needle pair for the microfabrication process. We provide references for the materials we used. However, the fabrication process can also be performed with materials from other suppliers (e.g. Fibreguide industries or Beckman Coulter).

**Movie S1 in File S1.** Movie S1 presents the monolayer formation of MCF10A cells within first 15 hours culture with adjusted flow of culture media. Cells during mitotic detachment are capable to maintain in the channel and adhere.
