# Supplementary material for: Facile Bench-Top Fabrication of Enclosed Circular Microchannels Provides 3D Confined Structure for Growth of Prostate Epithelial Cells
Source: PLoS One. 2014 Jun 19;9(6):e99416. doi: 10.1371/journal.pone.0099416 (PMC4063722; doi:10.1371/journal.pone.0099416)
Supplement: Movie S1 — Presents the monolayer formation of MCF10A cells within first 15 hours culture with adjusted flow of culture media. (DOCX) [file pone.0099416.s002.docx]

**Movie S1 in File S1.** Movie S1 presents the monolayer formation of MCF10A cells within first 15 hours culture with adjusted flow of culture media. Cells during mitotic detachment are capable to maintain in the channel and adhere.
